# O04 A novel, fast, accurate, cost-effective antimicrobial susceptibility test for urine samples

**DOI:** 10.1093/jacamr/dlac133.004

**Published:** 2023-01-19

**Authors:** Lucy Bock

**Affiliations:** UK Health Security Agency, UK

## Abstract

**Objectives:**

To show that iFAST (impedance-based fast antimicrobial susceptibility test) can give accurate susceptibility results for urinary tract infection (UTI) samples in less than 5 h.

**Background:**

UTIs are one of the most common forms of infection and reasons for antibiotic prescribing. Empirical prescribing without reference to antimicrobial susceptibility tests (ASTs) is common due to the high incidence, burden of symptoms and the length of time needed for an AST (24–48 h). However, untreated UTIs can lead to complications and in severe cases to bacteraemia and death. In addition, resistance to frontline antibiotics is increasing, leading to increased treatment failure. There is, therefore, a need for a fast, accurate and cost-effective AST for urine samples.

Bacterial impedance cytometry can measure the opacity and electrical size of individual cells by measuring the change in the electrical current as single cells flow between micro-electrodes. Bacteria treated with antibiotics change their electrical size and opacity compared with untreated cells. This change is visible on the iFAST within 2 h of exposure to above MIC of antibiotics, thereby enabling detection of cells susceptible to breakpoint concentrations of antibiotics. The iFAST technology and workflow can fit into current diagnostic laboratory practice.

**Methods:**

Fifty-eight *Escherichia coli* and *Klebsiella pneumoniae* strains were streaked onto standard agar plates and incubated at 37°C. After 2 h a streak of cells was inoculated into 0.9% saline. Cell count was measured using the iFAST and adjusted to 5 × 10^5^ cfu/mL. 100 μL samples were exposed to EUCAST susceptibility breakpoint concentrations of frontline UTI antibiotics in MH2: co-amoxiclav (2/8 mg/L), amoxicillin (8 mg/L), ceftazidime (1mg/L), cefalexin (16mg/L), ciprofloxacin (0.25mg/L), gentamicin (2 mg/L), nitrofurantoin (64mg/L), trimethoprim (4mg/L) or a no antibiotic control and incubated at 37°C. After 2 h, 60 μL of each culture were measured in the iFAST for 2 min and compared with the no antibiotic control, using percentage cell count and a 50% population contour. In parallel, a gold standard microbroth dilution MIC was carried out, according to EUCAST guidelines, on the cultures.

**Results:**

For the 441 tests conducted (58 strains in 8 antibiotics, nitrofurantoin is only recommended for *E. coli* treatment and was therefore disregarded for *K. pneumoniae*), overall concordance with the gold standard microbroth dilution MIC method was 95.2% for total cell count metric and 94.9% for cells in the population contour. The highest concordance was seen for ceftazidime (100% for both metrics) and amoxicillin (100 and 99% respectively), the lowest for trimethoprim (92% and 89% respectively). All non-concordances, except one strain with trimethoprim, showed an MIC of ±1 log_2_ (doubling dilution) different to the breakpoint concentration, which was used for iFAST. This is the normal technical variability of the gold standard method.

**Conclusions:**

iFAST has up to 100% concordance with current gold standard MIC methods and delivers the results in less than 5 h compared with 24–48 h for current ASTs. This novel, fast, accurate and cost-effective AST could increase evidence-based antibiotic prescribing, thereby improving patient treatment, decreasing risk of UTI complications and overall decreasing antimicrobial resistance development.

